# Understanding fake news during the Covid-19 health crisis from the perspective of information behaviour: The case of Spain

**DOI:** 10.1177/0961000620949653

**Published:** 2020-09

**Authors:** Michela Montesi

**Affiliations:** Library and Information Science Department, Universidad Complutense de Madrid, Spain

**Keywords:** Fake news, Covid-19, Spain, information behaviour, cognitive authority, affective authority, interactivity, civic values

## Abstract

The health crisis brought about by Covid-19 has generated a heightened need for information as a response to a situation of uncertainty and high emotional load, in which fake news and other informative content have grown dramatically. The aim of this work is to delve into the understanding of fake news from the perspective of information behaviour by analysing a sample of fake news items that were spread in Spain during the Covid-19 health crisis. A sample of 242 fake news items was collected from the Maldita.es website and analysed according to the criteria of cognitive and affective authority, interactivity, themes and potential danger. The results point to a practical absence of indicators of cognitive authority (53.7%), while the affective authority of these news items is built through mechanisms of discrediting people, ideas or movements (40.7%) and, secondarily, the use of offensive or coarse language (17.7%) and comparison or reference to additional information sources (26.6%). Interactivity features allow commenting in 24.3% of the cases. The dominant theme is society (43.1%), followed by politics (26.4%) and science (23.6%). Finally, fake news, for the most part, does not seem to pose any danger to the health or safety of people – the harm it causes is intangible and moral. The author concludes by highlighting the importance of a culture of civic values to combat fake news.

## Introduction

The Covid-19 pandemic of 2020 is leaving a profound wound in our society, and many think that our lives will never be the same again, with implications at all levels, including for library and information services. The avalanche of fake news and hoaxes that has accompanied the health crisis since its very beginning has converted an information issue into a topic of public opinion and debate, with pressure on the library community to give a satisfactory answer to the problem of how to recognize truthful and useful information (Xie et al., 2020). Explicit actions against disinformation have been taken since the 2016 electoral campaign for the US presidency, often in the form of guidelines and recommendations, whilst the library community has been debating about possible solutions to a problem that, Sullivan (2019) argues, we do not yet fully understand. So far, libraries have responded by reaffirming traditional library values and, as an immediate solution to what has been called an ‘infodemic’ (Marquina, 2020), the International Federation of Library Associations and Institutions (2020) updated its eight-step ‘How to spot fake news’ checklist on 16 March 2020, recommending additionally the exercise of critical thinking as an essential competence in media literacy.

The novelty of this new avalanche of fake news goes hand in hand with the novelty of the health crisis caused by the Covid-19 pandemic, which has converted fake news and information into a matter of social concern. Many social actors have contributed to a heated debate that has paralleled the health crisis, including the Spanish National Police (2020), which, on 27 March 2020, announced on its website the publication of a ‘Guide against fake news’. This guide, in the style of the International Federation of Library Associations and Institutions’ directions, recommends, among other strategies to check the veracity of information, relaunching Google searches, comparing the information found, being suspicious, verifying the author and to avoid sharing. Apart from the police, since March 2020 many Spanish professionals from different sectors have intervened to address the issue and encourage the population to break the chain of dissemination of clearly adulterated news. In his blog, the psychologist Soler Sarrió (2020) recommends Googling possible fake news and applying common sense. According to Soler Sarrió (2020), fake news aims to provoke fear and panic among the population, while Borondo (2020), from the newspaper *El Correo*, stresses that it cannot only cause Internet saturation, but could even put lives at risk. From the University of Barcelona, Vincent (2020) highlights the manipulative purposes of fake news, which seeks to scare, confuse and fuel divisions among the population, encouraging distrust of information from the government and other official sources. Emotional manipulation has been highlighted by Newtral (2019) as well, a journalistic website that is devoted to selecting and filtering information. However, the real social implications of the problem emerged from a survey by the Centro de Investigaciones Sociológicas published on 15 April. A sample of 3000 Spanish citizens was asked whether fake news should be prohibited and only official sources on the pandemic be permitted, and 67% of the respondents agreed that ‘it would be necessary to limit and control the information, establishing only one official source of information’. This caused protests against an alleged attack on the freedom of the press (Marcos, 2020), though it also threatens the library principle of unfettered access to information (Sullivan, 2019).

Although it is questionable whether fake news alone can generate division and social unrest, since, according to some sources, they would rather thrive on it and proliferate it in times of difficulties (Tandoc et al., 2018), it clearly introduces manipulative intentions in the consumption of information. Its purposes are both financial, seeking to increase the number of visits and clicks and consequently advertising revenues, and ideological, usually discrediting certain ideas and people in favour of others (Bakir and McStay, 2018; Tandoc, 2019). Lazer et al. (2018: 1094) define ‘fake news’ as ‘fabricated information that mimics news media content in form but not in organizational process or intent’, which differs from both ‘misinformation’ – that is, false or misleading information – and ‘disinformation’ – that is, false information that is disseminated intentionally to deceive people. Bernal-Triviño and Clares-Gavilán (2019), citing the European Commission, indicate that it would be more appropriate to speak of ‘disinformation’ because the term ‘fake news’ has been used to discredit the critical stance of certain information media that published truthful information. According to Bakir and McStay (2018), disinformation consists in deliberately creating and disseminating false information, while misinformation is the practice of those who, without being aware, disseminate false information – a phenomenon that has been little studied, the authors explain. According to Tandoc (2019), fake news can be considered a type of disinformation, whose main features include falsity, the intention to deceive and the attempt to look like real news. Rubin (2019) reiterates that the difference between misinformation and disinformation is intentionality, with both behaviours being supported by the highly technological affordances of our society. Social networks and online communication, together with the financial reasons mentioned above, are the basic foundations for the dissemination of false news (Blanco-Herrero and Arcila-Calderón, 2019). According to Rubin (2019), who applies an epidemiology-based model to the spread of fake news, social networks act as a means of transmission of the pathogen – the false news – whereas information-overloaded readers, with little time and without the appropriate digital skills, are the carriers. The warnings of the World Health Organization (2018: 26) go along the same lines, and this institution has been speaking of epidemics of rumours or an ‘infodemic’ in reference to ‘the rapid spread of information of all kinds, including rumours, gossip and unreliable information’, as a new threat to public health. Among the other motivations for spreading disinformation, Bakir and McStay (2018) underscore the affective dimensions of fake news, which rouses strong emotions, such as outrage, and takes advantage, among the other characteristics of online communication, of anonymity. Tandoc (2019), in order to explain people’s reasons for believing in fake news, discusses ‘confirmation bias’, or the inclination to believe in information confirming pre-existing beliefs, and ‘selective exposure’, or being exposed to content and information sources that are more attuned to one’s pre-existing attitudes and interests. However, according to Pennycook and Rand (2019), who measured the propensity to engage in analytical reasoning in a sample of 3446 participants who had been exposed to a set of fake and real news items, it was the participants’ willingness to engage in analytical thinking rather than confirmation bias that may have explained the difference in their ability to discern fake news from real news. In this study, analytical thinking allowed the participants to reject or disbelieve even politically concordant fake news articles.

### Fake news and everyday information behaviour

A lot has been written during the Covid-19 crisis of 2020 in an attempt to fight against disinformation. An important part of the research has focused on the analysis of all kinds of information spread via social media (Cinelli et al., 2020; Ferrara, 2020; Singh et al., 2020), whilst others have suggested interventions for improving news and science literacy as empowering tools for users to identify, consume and share high-quality information (Vraga et al., 2020b). The present contribution aims to understand the phenomenon of fake news from the perspective of information behaviour, pointing to uncertainty as a notable emotion in the context produced by the Covid-19 health crisis. All models of human behaviour in the consumption of information emphasize uncertainty as the factor that triggers the search for information itself, although traditionally it has been conceptualized more as a cognitive than an emotional trigger, at least in certain literature that has underscored the attributes of individuals above context and sociocultural frameworks in the study of information behaviour (Pettigrew et al., 2001). Since the 1990s, information behaviour has been studied in the framework of communicative processes and in connection with contextual factors of a social, cultural and ideological order, among others, including values and meanings (Pettigrew et al., 2001). The evolutionary perspective of Spink and Cole (2004) also refers to the environment or context when they point to the ability to obtain and exchange information as intimately linked to human survival. In the theory of Spink and Cole (2004), a behaviour of a constant searching for and collection of information from the environment, together with the architecture of the brain, has allowed the adaptation and survival of human beings. Human beings have been collecting and seeking information constantly, and not always consciously, in order to adapt to their environment and survive. From this perspective, information-related behaviour appears as an instinct, not always conscious, and a basic need of all human beings. Applied to the situation produced by the Covid-19 crisis, it can be said that the great uncertainty and the strong emotional charge regarding health, economic and social issues have created a heightened need for information as a strategy to cope with and adapt to an unusual and unexpected situation.

Uncertainty as well as other emotions have been given attention in the study of information behaviour as influencing factors that interact with cognitive factors. In the Kuhlthau (1991, 2005) model, emotions such as uncertainty, anxiety, optimism or worry fluctuate according to the different stages of the information-search process, accompanying the respective cognitive and decision-making processes. Nahl (2007) describes the synergy between cognition, emotions and the sensorimotor system in interactions with information technologies, explaining that adapting to environments with high information density implies a ‘load’ in all three dimensions. In Nahl’s (2005a, 2005b) theory of affective load in human information behaviour, affective processes interact with cognitive processes, providing the energy and motivation necessary to adapt to information technologies, for example, or regulating certain decisions, such as those regarding whether to use the information or not. Even in these models where emotions and other non-cognitive factors are assigned a role in information behaviour, decisions are made at the level of thinking and cognition. However, information decisions can also be made based on non-rational criteria and guided by emotions, corporeality and affect (Montesi and Álvarez Bornstein, 2017). These non-rational factors guide people’s judgement about the information they consume on a day-to-day basis and in situations of a lack of information and knowledge, which occur either because people enter specialized fields or because science and experts cannot always provide all the answers, as in situations of conflict between different sources of information – a phenomenon that has been studied in health information (Montesi, 2019).

In the initial stages of the Covid-19 crisis, and even later, experts and science were not able to provide all the answers that society expected. At the same time, uncertainty and the need for information were great, creating an important information gap in which other sources of knowledge came into play. Research on the search for health information teaches us that the information of health professionals, endorsed by health authorities, is usually complemented by what is called ‘experiential knowledge’ – that is, knowledge acquired as a consequence of experience (either personal experience or other people’s experience) in relevant situations (Montesi, 2019). This type of knowledge is usually exchanged when interacting with people (also on social media) and is closely related to social support, as it contributes to explaining and attributing meaning to the experiences that are being lived (Barbarin et al., 2016; Rubenstein, 2015). Experiential knowledge is especially valuable when facing situations of uncertainty and adaptation, not only for individuals but also for communities. Baillergeau and Duyvendak (2016) argue that ‘experiential knowledge’, as an alternative to expert knowledge, can guide policy responses in situations of high levels of uncertainty, specifically in the field of mental health policies. An important role is also recognized for experiential knowledge in climate change adaptation policies, where ‘local knowledge’ or ‘indigenous knowledge’, as it is referred to in this area, covers all the knowledge developed over a considerable period of time and shared by a community with respect to a specific locality. By its nature, local knowledge concerns adaptation mechanisms to changing environments, for both climatic and other factors, at the household and community levels (Naess, 2013). Experiential knowledge, as an alternative to official and authoritative knowledge from health systems, contributes to people being capable of making decisions about their health, and health literacy is, according to Samerski (2019), a social practice based on different sources and forms of knowledge, co-produced within the framework of social relations. Despite the fact that it can guide in situations of uncertainty and adaptation, and that it empowers people to manage their health, experiential knowledge is still not recognized as evidence, and expert knowledge continues to condition the discourse and definitions of health and social problems (Popay, 2018).

In short, during the Covid-19 health crisis, fake news has been spread in a context of great uncertainty and emotional load that has generated a heightened need for information as a mechanism for understanding and adapting to an unprecedented and threatening event. The urgency of the situation has pushed us to look for quick solutions as a response to fake news, misinformation and disinformation – such as guidelines in bulleted points or automatic checks via Google or other information ‘authorities’ such as FactCheck.org (Bernal-Triviño and Clares-Gavilán, 2019) – and a significant proportion of the Spanish population surveyed by the Centro de Investigaciones Sociológicas (2020) supported the idea of a single official information source. In the end, the wide spread of false news calls into question all the knowledge that is produced outside official communication channels, as well as the rights of citizens to exercise their judgement on the information they consume. Delegating decisions on information to authorities, whether health, scientific or others, is a common choice to assess trustworthiness and credibility, but during the Covid-19 crisis it has been more pronounced and potentially harmful, as it might suppress all other sources of information, not only news media. Saunders and Budd (2020) remind us that, in library education, the credibility of information sources is assessed by looking at the credentials of who has produced them and their publication track record, reinforcing existing biases in the production of knowledge, including gender bias, in favour of institutionalized knowledge, and underestimating the need to train future library and information professionals on the evaluation of scientific information and contents. In other words, rather than checklists or predefined recipes for fighting fake news, misinformation and disinformation, it is necessary to develop critical thinking skills to apply to the content and information to which we are exposed on a daily basis. Vraga et al. (2020a) propose an initiative of news literacy combined with expert corrections of misinformation. However, fact-checking initiatives intended to debunk fake news might also fail as a strategy, as most people might ignore evidence or even continue to hold onto their pre-existing ideas, even after exposure to it (Tandoc, 2019). In addition, monopolizing control over information might have undesirable consequences and pave the way for censorship (Sullivan, 2019). It is also important to defend the legitimacy of information and knowledge that is acquired and shared outside institutionalized settings and as a result of experience, as it might be a meaningful complement to scientific knowledge, according to the vast research on health information behaviour. On the basis of these assumptions, better knowledge of disinformation is needed not only to improve research into the automatic detection of anomalous information (Zhang and Ghorbani, 2020), but also to avoid fast and potentially harmful solutions. Such research addresses the following questions in particular: Does fake news rely on experiential knowledge? How does it manage to appear ‘authoritative’ to people who contribute to its dissemination? To what degree is it harmful? Characterizing and understanding false news can help us to recognize and reject it based on the exercise of critical thinking. With this objective, in this work a set of false news items spread during the Covid-19 crisis in Spain is analysed.

## Methodology

The sample of fake news analysed was obtained from the Maldita.es website, a project that is part of the International Fact-Checking Network initiative, which has been collecting fake news since 2017 (Bernal-Triviño and Clares-Gavilán, 2019). The methodology, on the basis of which it is established whether a news item is considered false, is described on the website (Maldita.es, 2018); it focuses mainly on the verification process while omitting details regarding the news selection process. All the fake news is discussed thoroughly on Maldita.es and refuted on the basis of additional public sources. In total, 242 fake news items were classified. As of 6 April 2020, when the analysis of fake news was initiated, the site had collected 393 news items that had been produced during the Covid-19 health crisis alone. By the end of April, when the classification reached its end, the collection numbered almost 500 items and was continuing to grow. The fake news on Maldita.es does not follow a chronological order, and consecutive chunks of news were classified at the beginning, at the end and in the middle of the series during the month of April. The fake news collected from Maldita.es on 6 April 2020 included all 46 false news reports about Covid-19, with the exception of three, which the Intelligence Centre against Terrorism and Organized Crime (2020) of the Ministry of Internal Affairs collected in a report that was published on 17 March 2020, providing a certain guarantee of coverage of the main hoaxes that were spread in the course of the 2020 health crisis.

In order to classify the news against a set of quality criteria, I first looked at the literature on health-information seeking and the criteria that come into play when evaluating health information. Among the elements that influence the quality of health information on the Web, Sbaffi and Rowley (2017) highlight the website’s design, the authority of the person/institution responsible for the site and the possibility to make contact, as well as the availability of other channels of interaction. Similarly, Zhang et al. (2015) emphasize the importance of factors related to web design – in particular, interactivity and the possibility of exchanging information with other people, expansion through social media, the presence of an internal search engine, multimedia documents and the availability of explicit disclaimers. At an operational level, the work of Sun et al. (2019) was also taken into account. They define quality as ‘fitness for use’ – that is, quality information must serve the user’s needs – and, in order to ‘measure’ it, Sun et al. (2019) differentiate ‘criteria’, or rules, that people apply to information objects to determine their value – reliability, experience, objectivity, transparency, popularity or understandability, among others – from ‘indicators’ – that is, perceptible elements of the information objects that allow their quality to be determined. The set of indicators that Sun et al. (2019) propose is deployed in three broad sections. Indicators related to content cover both the information and the presentation, and include aspects such as themes and concepts, writing, presentation, references, authorship, audience, current events and the presence of advertisements. Design-related indicators refer to the appearance and structure of the website or application, and the possibilities of interaction it provides. Finally, the indicators related to the source include who creates, hosts and distributes the content, and the site typology, as well as its popularity and other systems’ recommendations. Unfortunately, many of these indicators are not applicable in this research. Usually, fake news is spread outside of a website’s context and as direct falsifications of official documents or informal communication devices such as tweets or social media accounts, among others. For this reason, many information literacy programmes addressing fake news may be ineffective (Sullivan, 2019), whilst artificial intelligence can be used to digitally manipulate video and audio files to deliver what has been called ‘deep fakes’ (Tandoc, 2019). From this literature on the evaluation of health information, I have retained two concepts – authority and interactivity – which I have measured as explained below.

### Cognitive authority and affective authority

The concept of cognitive authority is one of the most studied in information-related behaviour (Rieh, 2002). As Neal and McKenzie (2011) explain, currently the ‘cognitive authority’ of an information source is conceived as the result of social practices that allow a certain community to negotiate what counts as an authorized source of information. Citing the 2016 *Framework for information literacy for higher education* of the Association of College and Research Libraries, Saunders and Budd (2020) add that cognitive authority is not only constructed, but also contextual, depending on the information needs of the situation, and that it covers not only traditional indicators of authority, such as subject expertise and societal position, but also lived experiences, such as those shared on blogs or social media. With reference to experiential knowledge, a second affective dimension of authority comes into play, which builds on the subjective properties of the information being shared, such as appropriateness, empathy, emotional supportiveness and aesthetic pleasure (Neal and McKenzie, 2011). As Lynch and Hunter (2020) point out, cognitive authority alone might be insufficient to deal with misinformation, and reflection on each individual’s social and emotional factors might cast light on the dynamics of affective authority. According to Montesi and Álvarez Bornstein (2017), from an affective point of view, decisions about information also rely on non-rational and not always conscious indicators originating from senses, emotions and intrapersonal knowledge, especially when decisions need to be made in situations of conflict among different information sources and points of view. Following Neal and McKenzie (2011), the affective authoritativeness of experiential information sources rests on the account of the experience itself and its details, the similarity of the experience narrated with the reader’s experience and, finally, the ability to comfort or inspire that personal experience provides over mere information. Although they do not explicitly mention the affective dimension of authority, Hirvonen et al. (2019), who analyse a health forum for young women, add that the reliability of experiential knowledge is judged on the grounds of an array of elements, ranging from data related to the author to the way of arguing and tone (including language and style), the veracity or coherence with the reader’s prior knowledge, and verification through comparison of various sources. It is important to differentiate this ‘affective authority’ of the content being disseminated from the affective authority of those who disseminate information, including false news, since, as Montero-Liberona and Halpern (2019) point out, much false health news comes precisely from acquaintances and trusted people.

In the classification of fake news, I have taken into account aspects that were relatively easy to detect which could allow a classification out of context, pointing to properties of the news that might have convinced the reader. Specifically, and after a first informal browsing of the set of news items being classified, I have tried to operationalize the above concepts of cognitive and affective authority in the following way. Regarding cognitive authority, it has been determined whether the information provided in the fake news (1) derived from direct and first-hand experience, justified in the way the studies mentioned above describe (experiential knowledge); (2) relied on subject expertise without institutional endorsement or other types of endorsement (the name, surname and professional qualification were provided and no more); or (3) derived from subject expertise endorsed by an institution or a publication track record. Additionally, I coded (4) direct falsifications and (5) the total absence of indicators of cognitive authority.

Capturing the affective component of authority based on the news itself and out of context is more difficult. In order to be able to locate cues of affective authority, I exploited, on the one hand, the concepts of fake news being used to discredit opponents (Bakir and McStay, 2018; Tandoc, 2019) and of conflict among information sources as a condition for making ‘affective’ decisions about information (Montesi and Álvarez Bornstein, 2017). On the other hand, I used some of the strategies described in Hirvonen et al. (2019) to weigh experiential knowledge, in particular those pertaining to language and the comparison of sources. As a result, the following elements have been recorded: (1) whether the news discredited people, ideas or movements in favour of others that were supposedly common to the recipients of the hoax; (2) if coarse or offensive language was used; and (3) if additional sources were mentioned or opportunities for further study were offered. I understood that the comparison of sources denotes a legitimate and genuine intention to transfer the knowledge acquired through personal experience. These were considered the easiest and most objective elements to detect, bearing in mind that it was not always possible to access the primary source.

### Interactivity

As mentioned previously, the literature on health-information seeking on the Web points to interactivity as an important element to consider when evaluating information. According to Sun et al. (2019), interactivity is all the possibilities within a site to communicate with the system or other users, and to adjust content to consumer needs. This broad definition covers a varied range of features, such as internal search functions, devices for commenting on content and allowing user input and information exchange, multimedia content or personalization tools. The breath of the concept makes it difficult to use interactivity in fake news classification, especially if we consider that fake news is often disseminated outside of a website and that it is often ephemeral in nature. Indeed, the literature dealing with the topic of interactivity supports a complex conception of it. Oh and Sundar (2015) differentiate ‘modality interactivity’, or ‘tools or modalities available on the interface for accessing and interacting with information’ (215), from ‘message interactivity’, or ‘the degree to which the system affords users the ability to reciprocally communicate with the system’ (217). Yang and Shen (2018) employ a meta-analysis to determine the effects of interactivity on cognition (as knowledge elaboration, information processing and message retrieval), enjoyment, attitude and behavioural intentions. The inconsistent conclusions they reach, pointing to a positive effect in all dimensions except cognition, suggest that interactivity might influence users’ experience via two different routes: a cognitive route and an affective route. Yang and Shen (2018) conclude that, even if web interactivity does not support user cognition, it might raise affective responses, such as enjoyment, developing as a consequence favourable attitudes and behavioural intentions. According to Oh and Sundar (2020), actions such as clicking, swiping and dragging allow users to exert greater control over the content and to feel absorbed and immersed cognitively and emotionally in it. Without systematically processing the website’s message, users may express a more positive attitude towards its content by feeling absorbed in interactive devices.

Although it might be difficult to identify interactivity clearly, it appears to be related to the affective dimension of authority that I discussed earlier, and it is therefore pertinent to devote some attention to it in this research, even if with limitations. Basing the analysis exclusively on the news does not allow us to understand user perspectives on interactivity (Sohn and Choi, 2014), and it is impossible to measure for fake news features such as search capabilities or personalization tools. All fake news is, to a certain extent, interactive, as it is precisely thanks to certain interactivity features that it goes viral. In this classification, I adopted a simple conception of interactivity and recorded whether the interactivity supported was simply one-click interactivity (forward, share, hashtag or like) or interactivity that at least allowed a certain degree of interaction and dialogue through the comments option. One-click interactivity covers all audio or text messages, images and videos sent via WhatsApp, television programmes, and certain news published in web magazines and media that did not allow commenting. When it was not possible to determine whether the news allowed commenting, interactivity was coded as ‘impossible to determine’.

### Themes

I have classified fake news into three themes: politics, science and society. Although it tends to be predominant in politics, health fake news is also common (Montero-Liberona and Halpern, 2019) and it was expected that, in the Covid-19 crisis, it was being widely disseminated. In this study, health news was classified under science. A previous analysis of fake news collected from Maldita.es revealed that, out of 568 news items, most had politics as the main theme (35%), whilst the rest were distributed among people (15%), immigration and racism (12%), gender (10%) and science (9%) (Bernal-Triviño and Clares-Gavilán, 2019). I decided to remove ‘people’ as a category because many fake news pieces specifically attack people in the world of politics and thus have a political intention.

### Potential danger for people’s security or health

According to Tandoc et al. (2018), in most cases, fake news is ignored and does not lead readers to further action, except for in some anecdotal cases, although concerns have been expressed about its ability to influence election results and confuse readers. However, in some exceptional cases, it can lead to extreme episodes of violence (Tandoc, 2019). In addition, much fake news has a certain sense of humour – something that can convince us of its harmless nature. However, the real danger it can result in is unknown. Therefore, based on the information provided by Maldita.es and complementary information searches, I attempted to determine whether fake news could result in potential danger to people’s health or safety.

## Results

All of the news that Maldita.es had collected as fake was treated as such, with a few exceptions. Phishing emails were excluded, as usually they are not intentionally spread in the same way as fake news, and so were some clear mistakes, such as an audio message from a doctor recorded for her family when leaving a meeting, which had been spread virally. I understood that these cases were not false information. Maldita.es has also collected as fake news interpretations of information that are not always clear. This was the case, for instance, for the controversy about whether children were allowed a walk in Italy or not between the end of March and the beginning of April 2020. All of these cases were included in the 242 analysed cases but they were not classified. In what follows, descriptive statistics are presented for authority, interactivity, themes and potential danger. In some cases, the possible association of some news features with others was tested by applying the Chi-square test, such as the association between the use of offensive and coarse language and the theme of the news item. When the null hypothesis could be rejected, it is indicated in the text. The data was processed using Excel and IBM SPSS Statistics.

### Cognitive authority and affective authority

Table 1 shows that the sample of classified fake news presents, in most cases, no cues of cognitive authority. In more than half of the cases (53.7%), the information provided is not based on personal or professional experience. In 12.6% of the news items, the authors introduce themselves by giving their name, surname and professional qualification, but do not mention any institutional affiliation or other type of endorsement. Clear falsifications account for 19.2% of all cases. The falsified news included all the alleged declarations of well-known people – such as Bill Gates, Noam Chomsky or Pope Francis – counterfeit tweets or other social media content published by major news media outlets, the Spanish National Police, ministries, or other departments of local and national government. In only 10 instances (4.7%) did I find some type of endorsement. This was the case with journalists publishing incorrect information, which was often rectified in news media outlets by Members of Parliament or experts such as Thomas Cowan, the author of several books.

**Table 1.** Fake news classification based on authority criteria.

**Cognitive authority**		
Does the fake news provide cues of cognitive authority?	Number of fake news items	%
**Impossible to determine**	21	9.8
**Yes, based on documented first-hand experiences**	0	0.0
**No**	115	53.7
**Yes, based on professional expertise with no affiliation**	27	12.6
**Yes, based on professional expertise supported by** an **affiliation or publication track record**	10	4.7
**It is a falsification**	41	19.2
**Total**	214	100
**Affective authority**		
Does the fake news compare different information sources? Does the fake news refer to additional information sources?	Number of fake news items	%
**Impossible to determine**	40	18.7
**Yes**	57	26.6
**No**	117	54.7
**Total**	214	100
**Does** the fake news **discredit people, ideas or movements?**		
**Impossible to determine**	25	11.7
**Yes**	87	40.7
**No**	102	47.7
**Total**	214	100
**Does t**he fake news **use coarse or offensive language?**		
**Impossible to determine**	51	24.4
**Yes**	37	17.7
**No**	121	57.9
**Total**	209	100

Regarding affective authority, 40.7% of the news discredits people, ideas or movements, whilst 17.7% does so using coarse or offensive language. Although in 26.6% of the cases other sources are mentioned or referred to, often these sources do not exist as a result of falsifications or their removal. Even so, this strategy may be enough to confer a certain affective authority on the news.

### Interactivity

In 57.9% of the cases, the hoaxes use one-click ‘interactivity’, such as forward, share or hashtags. In 24.3% of the cases, comments are also allowed, especially from Twitter accounts or for YouTube videos. Many of the comments had been disabled on the date I accessed the news (especially on YouTube). Where the comments had not be disabled, often it was mentioned that the news was false or incorrect (see Table 2).

**Table 2.** Classification of fake news according to interactivity criteria.

Does the design allow interactivity?	Number of fake news items	%
Impossible to figure out	37	17.3
No	1	0.5
It allows one-click interactivity (forward, share, hashtags or likes)	124	57.9
It allows commenting	52	24.3
Total	214	100

One-click interactivity occurs more frequently when the hoax is a direct falsification (Pearson’s chi-squared = 7.739, *df* = 1, *p* < 0.005) and when it does not compare or refer to additional information sources (Pearson’s chi-squared = 7.099, *df* = 1, *p* < 0.008).

### Themes

In the classification by theme, society accounts for 43.1% of all cases, followed by politics (26.4%) and science (23.6%). All the fake news published in the category of science concerned health topics and, despite having all been published during the Covid-19 health crisis, which should have emphasized health over the other categories, science accounted for less hoaxes than politics and society. The most common topics of the health fake news classified in the category of science included home remedies for treating, preventing or diagnosing Covid-19; explanations about the origin of the virus, including the names of scientists allegedly responsible for the pandemic; vaccines; or advice regarding masks and hygiene procedures to avoid infection. Popular news in politics often targeted members of the government and, secondarily, other politicians, who were accused of having preferential access to the health system’s resources, breaching the lockdown or underestimating the impact of the pandemic based on the alleged evidence. All measures that limited the freedom of citizens were often misinterpreted and inflated. Finally, society fake news was concerned with well-known people and companies, especially supermarket chains and social media companies; often had a racist background; showed images of animals in deserted urban scenes; and sometimes had an ironic tone (see Table 3).

**Table 3.** Classification of fake news according to theme.

Themes	Number of fake news items	%
Science	51	23.6
Politics	57	26.4
Society	93	43.1
Various themes	15	6.9
Total	216	100

More frequently than society or science fake news, politics fake news used coarse or offensive language (Pearson’s chi-squared = 42.598, *df* = 2, *p* < 0.000) and discredited people, movements or ideas (Pearson’s chi-squared = 59.603, *df* = 2, *p* < 0.000).

### Potential danger for people’s security or health

As can be seen in Table 4, it is clear that the vast majority of the news does not imply any danger to people’s health or safety, since only 17 of the 214 news items that could be classified according to this criterion represent certain types of danger either for public safety or people’s health. Among the cases that were classified as potentially dangerous for health, meaningful examples include a supposed vaccine against Covid-19 that could be used to manipulate the population, advertisements showing people offering to be infected with the virus, or the alleged minor vulnerability of smokers to Covid-19. On the side of fake news that was potentially dangerous for the security of people, examples include all news dealing with the impulsive behaviour of people rushing to supermarkets and stockpiling food or other commodities, which could invite people to reproduce similar behaviour.

**Table 4.** Classification of fake news according to potential danger.

	Is it a threat to people’s health?		Is it a threat to people’s security?	
	Number of fake news items	%	Number of fake news items	%
Impossible to figure out	18	8.4	18	8.4
Yes	9	4.2	8	3.7
No	187	87.4	188	87.9
Total	214	100	214	100

## Discussion and conclusion

In this research, a sample of fake news items collected by the Maldita.es project during the Covid-19 health crisis in Spain was classified according to the criteria of authority, interactivity, theme and potential danger. With regard to authority, no single news item was based on personal first-hand experience and only 4.7% of the pieces were based on professional expertise supported by an affiliation or a publication track record. More than half of the sample (53.7%) did not present any elements whatsoever that permitted mention of cognitive authority. In the rest of the cases, the information provided was either a clear falsification (19.2%) or came from alleged professionals who, with their name, surname and professional qualification but no other endorsement, intended to contribute their knowledge (12.6%). From the perspective of affective authority, hoaxes created ‘complicity’ with their recipients through strategies of discrediting people, ideas or movements (40.7%), often using coarse or offensive language (17.7%), pointing to the connection of affective responses with situations of polarization or conflict among information sources. Both strategies were related to fake news whose main theme was politics. Additionally, in more than a quarter of the cases (26.6%), the fake news used a strategy of apparent transparency by comparing or referring to additional information sources, which probably helped to gain the trust of the recipients.

As for interactivity, 24.3% of the fake news items allowed comments and, in theory, an exchange of information with the author of the news or other people, while 57.9% only allowed some type of one-click interactivity, such as like, share or forward. For 17.3% of the news items, it was impossible to determine whether they supported commenting. One-click interactivity was related to falsifications more often than expected, whilst commenting was related to comparison or reference to additional information sources more often than expected, which means that interactivity features appeared to be related to different strategies of constructing authority. When authority rests on falsified author credentials, interactivity tends to be minimal – just enough to allow the spread of the news. When authority is built through a strategy of comparison and references to additional information sources, as usually happens when experiential knowledge is shared, it might support comments and, with these, a certain degree of participation. It is important to stress that most often the additional or referenced sources are also false or do not exist. What I am counting here is the act of referencing and supporting the news. Research into interactivity has not be conclusive on the cognitive effects of physical and click-based interactivity (Yang and Shen, 2018), though apparently it can create significant changes in cognitive and emotional processing, as well as in attitudes and behaviours related to the information processed (Oh and Sundar, 2020). Social media per se and their interactive features do not always support real dialogue and communication, especially when they are used with political purposes (Pérez Curiel and García-Gordillo, 2018), and even if likes or shares are often taken as indicators not only of interactivity and engagement but even of bi-directional and participative communication (Sáez-Martín and Caba-Pérez, 2018). It is important to remember that, in the context of the Covid-19 crisis, the need to consume information might have been much higher than usual, and even simple one-click actions might have allowed some kind of engagement and participation in information exchanges. Cinelli et al. (2020: 9), who looked at 8 million comments and posts over a time span of 45 days on five social media platforms during the Covid-19 crisis, meaningfully observe that the spread patterns of questionable information do not differ from those of reliable information, concluding that ‘information spreading is driven by the interaction paradigm imposed by the specific social media or/and by the specific interaction patterns of groups of users engaged with the topic’. Future research should pursue a clearer definition of all these concepts and investigate how interactivity cooperates in supporting authority, on the one hand, and communication and participation, on the other.

Fake news items with society (43.1%) as the theme outnumbered those on both politics (26.4%) and science (23.6%). It was surprising that science, which covered health, was the least popular subject in the middle of an unprecedented health crisis. Health and politics discussions during the crisis might have followed different patterns, as Ferrara (2020) explains on the basis of 43.3 million English tweets about Covid-19, concluding that tweets generated by bots were different from those generated by human users in that the former presented political connotations whereas the latter were concerned mainly with health and welfare issues. It might also be some feature of scientific information itself that explains this difference, such as the availability of valuable health information or the high level of specialization required to access and make use of scientific information, even in a manipulative way. Scientific information is also based on peer review, which is, to a certain extent, a participative process, leading to the agreement of what counts as evidence and reducing conflict and polarization.

Finally, the vast majority of fake news does not result in any danger to the health or safety of people, which can lead us to consider it as harmless. Indeed, some fake news is quite inoffensive. It does not cause any harm to claim that deer are trotting around in a Spanish village when the video was actually shot in Italy, because the images remain astonishing and worth sharing for their aesthetic value. However, taking as evidence the affective authority mechanisms mentioned above, there is some damage that disinformation might cause, which is not only intangible but also of a moral nature. Sullivan (2019) insists that the problem is not the existence of disinformation itself but what it might do to our minds. The literature on the subject emphasizes that consumers of information tend to prefer information that confirms their pre-existing attitudes and visions of the world, and give preference to information that is gratifying over that which calls into question their expectations (Lazer et al., 2018; Montero-Liberona and Halpern, 2019). This phenomenon has been called ‘confirmation bias’ (Tandoc, 2019). However, I contend that this inclination towards the familiar can be conditioned by previous or prior knowledge – that is, all the information we have stored as a result of our experiences and as members of a certain society, and that we need in order to process and make sense of new information (Renkema and Schubert, 2018). In a certain sense, it is to be expected that we prefer what is coherent with our prior knowledge and can be made sense of, even if our mental frameworks can sometimes distort facts according to socially and culturally shaped schemas of the world. Perhaps, instead of correcting this natural inclination of human beings, we should correct the very concept we have of knowledge and start to include, apart from facts, values and meanings, as research into climate adaptation suggests (Bremer and Meisch, 2017). If fake news is an indicator of social tension and divisions, as mentioned above following Tandoc et al. (2018), what it is showing, by discrediting without foundation people, ideas and movements, falsifying, and using coarse and offensive language, is a failure of civic values in contemporary society. And this does not only affect those creating the fake news, but also all those who are contributing to its dissemination. It is not enough to combat fake news from a purely cognitive angle, recommending checklists and honest expert control (Rodríguez-Ferrándiz, 2019: 9), or rectifying and correcting misinformation through news literacy interventions (Vraga et al., 2020b). It is necessary to look for a solution within the complexity of human beings and our society that not only promotes critical thinking but also encompasses values and beliefs. Libraries are proposing to broaden the ideological spectrum of their collections, highlighting the pluralism of the society they serve (López-Borrull et al., 2018). However, Sullivan (2019) points to a tension in traditional library values that, on the one hand, aim to provide unrestricted access to information and, on the other, offer ‘epistemological protection’ by selecting information according to an unquestionable concept of quality. The solution to the apparently unsolvable problem of fake news probably requires a much deeper redefinition of values than simply making room for more pluralism and, according to the results of this study, affective nuances of knowledge and authority should be thoroughly explored and understood in order to take further steps in the fight against fake news.
